# Indications, Feasibility, and Safety of TST STARR Plus Stapler for Degree III Hemorrhoids: A Retrospective Study of 125 Hemorrhoids Patients

**DOI:** 10.3389/fsurg.2022.860150

**Published:** 2022-04-13

**Authors:** Jun Wei, Xufeng Ding, Jie Jiang, Lijiang Ji, Hua Huang

**Affiliations:** Department of Anorectal Surgery, Changshu Hospital Affiliated to Nanjing University of Chinese Medicine, Changshu, China

**Keywords:** degree III hemorrhoids, stapled hemorrhoidopexy, PPH stapler, TST STARR plus stapler, prolapse recurrence

## Abstract

**Background:**

Stapler hemorrhoidopexy (SH) has been widely accepted for hemorrhoids patients because of its low postoperative pain, but it is also associated with a high recurrence rate. The recurrence might be due to failure to completely remove the prolapsed tissue or insufficient removal capacity of the instruments. Removing more prolapsed tissue to reduce the recurrence is believed to benefit more severe prolapsed hemorrhoids patients.

**Methods:**

We evaluated the short- and long-term safety and efficacy in 125 hemorrhoids patients who underwent SH in 2013–2015. Eighty patients had prolapsed tissue less than half of the circular anal dilator (CAD) and underwent a procedure for prolapsing hemorrhoids (PPH), while the remaining 45 patients with hemorrhoid prolapse greater than half of the CAD were treated with a tissue selection therapy stapler stapled transanal rectal resection plus (TST STARR+).

**Results:**

There were no significant differences between the two groups in terms of operative time, hospitalization time, overall satisfaction or complications. At follow-up of up to 4 years after surgery, there was no significant difference in recurrence rates between TST STARR+ group and PPH group (5.2% vs. 4.7%, *p* < 0.05). The mean width and volume of the resected tissues were significantly larger in the TST STARR+ group than in the PPH group (4.8 vs. 2.9 cm, 10.2 vs. 4.4 cm^3^, *P* < 0.05).

**Conclusion:**

The TST STARR+ procedure can remove more hemorrhoidal tissue than PPH and it is better suited for patients with severe annular prolapsed hemorrhoids greater than half of the CAD. It has the advantages of convenient to operate, rapid recovery, fewer complications, and long-term satisfactory results.

## Introduction

Hemorrhoids are swollen, enlarged veins in the anal canal, with a prevalence of 39%, according to a study of patients with routine colorectal cancer screening (1). The main clinical manifestations of hemorrhoids are bleeding and hemorrhoid prolapse. Surgery is the main modality for treating degree III hemorrhoids (2–4). The procedure for prolapsing hemorrhoids (PPH) proposed by Longo in 1998 has become an alternative to conventional hemorrhoidectomy (CH) in selected patients (5). Several randomized, controlled trials have shown that stapled hemorrhoidopexy has the advantage of less postoperative pain and faster recovery than conventional hemorrhoidectomy, but it has a higher recurrence rate (6–9). To improve the clinical effect and reduce the recurrence rate, the Italian professor Naldin developed tissue selection therapy stapler stapled transanal rectal resection plus (TST STARR+), which is a high-volume device for the treatment of prolapsed hemorrhoids. Because its external diameter is 36 mm, and the anastomotic cavity volume is >35 cm^3^, more tissue can be removed under direct vision (10). The purpose of this study is to evaluate the feasibility, safety, and long-term outcomes of TST STARR+ in the treatment of grade III hemorrhoids compared with PPH.

## Materials and Methods

### Patients

To compare the difference in the amount of tissue removed, complications, and long-term prognosis between the TST STARR+ and PPH, we retrospectively analyzed 125 patients with degree III hemorrhoids, 45 in the TST STARR+ group and 80 in the PPH group, who underwent stapled hemorrhoidectomy from October 2013 to December 2015 in Department of Anorectal Surgery, Changshu Hospital of Traditional Chinese Medicine.

The indication for both procedures is patients with prolapsed hemorrhoids who have symptoms of prolapse and have failed conservative treatment. The inclusive criteria for this study were: (1) grade III hemorrhoids (Goligher classification); (2) aging 18 to 70 years old; and (3) absence of significant anal deformity. Patients with inflammatory bowel disease, perianal abscess, fistula, anal fissure, bowel abnormalities (constipation or incontinence), and coagulation disorders were excluded. The study was approved by the ethics committee of Changshu Hospital of Traditional Chinese Medicine. All of the operations were performed by a specialist of anorectal surgeon.

### Treatment

On the morning of surgery, an enema was performed with chitosan quaternary. The procedure was performed with endospinal anesthesia, and the patient was placed in the left lateral decubitus position. To obtain a better outcome by choosing the appropriate surgical approach, the size of the prolapsed tissue was assessed by circular anal dilator (CAD) after the patients were completely anesthetized. TST STARR+ surgery was used for tissue prolapse greater than half of the CAD, while PPH surgery was used for tissue prolapse less than half of the CAD. If bleeding from the anastomosis occurred intraoperatively, both surgical approaches would be stopped with 3-0 polyglycolic acid sutures, and the number of stitches used to stop the bleeding was recorded. The volume and width of the resected hemorrhoidal tissue were measured after surgery. The volume was calculated by immersing the tissue in a container filled with water and measuring the drainage. Postoperatively, patients routinely took stool softener or analgesic orally.

### Data Collection

The patients' age and sex, the volume and width of the resected hemorrhoidal tissue, the median length of surgery, the number of additional external hemorrhoidectomies, and the number of anastomotic hemostatic sutures were recorded.

Recurrent hemorrhoid prolapse after surgery was recorded as an efficacy indicator. The follow-up time points were 1 month and 12 months postoperatively. Follow-up was extended to 48 months to assess postoperative recurrence rates. Additionally, adverse events (AEs), such as urinary retention, bleeding and urgency, were recorded as safety indicators.

### Statistical Analysis

Data were analyzed using SPSS software, version 22.0 (SPSS Inc., Chicago, IL, USA). Considering normality, continuous data are shown as median and interquartile range (IQR) and analyzed using the Mann-Whitney U-test. Categorical data are presented as counts and percentages and analyzed with the chi-square test or Fisher's exact test. Statistical testing was two sided, and *P* < 0.05 was considered statistically significant.

## Results

### Characteristics of the Patients

A total of 125 patients underwent stapled hemorrhoidopexy, of whom 80 patients (52 men and 28 women) underwent PPH surgery, with an average age of 53.1 years old, and 45 patients (33 men and 12 women) underwent TST STARR+ surgery, with an average age of 51.7 years old. There were no statistically significant differences in age or sex between the two groups (*P* > 0.05).

The median width of the resected tissue in the TST STARR+ group was 4.8 cm, and the median volume was 10.2 cm^3^, while the median width of the resected tissue in the PPH group was 2.9 cm, and the median volume was 4.4 cm^3^. It was clear that, in both dimensions, tissue size in the TST STARR + group was larger than that in the PPH group (*p* < 0.05), and the TST STARR + operation could remove more hemorrhoids. There were no significant differences in the median length of surgery, the number of additional external hemorrhoidectomies, or the number of anastomotic hemostatic sutures (*P* > 0.05), as shown in Table 1.

**Table 1:** Characteristics of the patients.

**Group**	**TST STARR +**	**PPH**	***χ^2^*/*z***	***P***
Number	45	80	–	–
Surgery time(min)	25(7)	23/11	−1.873	0.061
Additional external hemorrhoidectomies	31(68.9%)	61(76.3%)	0.803	0.370
Tissue width (cm)	4.8(1.1)	2.9(0.8)	−9.253	0.000
Tissue volume (cm^3^)	10.2(3)	4.4(1.2)	−9.262	0.000
Hemostatic stitches	2(4)	3(3)	−0.138	0.890

*Surgery time, Tissue width (cm), Tissue volume (cm^3^), and Hemostatic stitches are presented as Median (IQR), and Additional external hemorrhoidectomies as N (percentage)*.

### Safety Assessment in 1 Month

No serious AEs were reported in either group. In the perioperative period (≤30 days), however, some patients reported minor AEs. A total of 14 patients developed urinary retention and required catheterization: 6 (13.3%) in the TST STARR+ group and 8 (10.0%) in the PPH group (χ^2^ = 0.322, *P* = 0.571). Six patients experienced a brief sensation of urgency: 3 (6.7%) in the TST STARR+ group and three (3.8%) in the PPH group (χ^2^ = 0.536, *P* = 0.464). Two patients in the PPH group (2.5%) experienced postoperative bleeding requiring reoperation interventions, which did not occur in the TST STARR+ group (χ^2^ = 1.143, *P* = 0.285), as shown in Table 2.

**Table 2:** Safety assessment at 1 month.

**Group**	**TST STARR +**	**PPH**	***χ^2^*/*z***	***P***
Number	45	80		
Urinary retention	6(13.3%)	8(10%)	0.322	0.571
Urgency	3(6.7%)	3(3.8%)	0.536	0.464
Minor bleeding	0(0%)	2(2.5%)	1.143	0.285

*Urinary retention, Urgency, and Minor bleeding are presented as N (percentage)*.

### Long Term Efficacy and Safety

To track patients' long-term efficacy, we performed follow-up visits at 12 months after surgery. At the 12-month follow-up, there was a substantial decrease in the number of patients with postoperative AEs in both groups. Regarding recurrence, 2 patients (2.5%) in the PPH group experienced recurrence of prolapse, and no patients in the TST STARR+ group reported recurrence of prolapse (χ^2^ = 1.143, *P* = *0*.285), as shown in Table 3.

**Table 3:** Follow-up at 12 months.

**Group**	**TST STARR +**	**PPH**	***χ^2^*/*z***	***P***
Number	45	80		
Minor bleeding	0(0%)	3(3.8%)	1.729	0.189
Anastomotic stenosis	0(0%)	2(2.5%)	1.143	0.285
Prolapse recurrence	0(0%)	2(2.5%)	1.143	0.285
Urgency	2(4.4%)	3(3.8%)	0.352	0.553
Satisfaction index	9(2)	9(3)	−1.406	0.160

*Minor bleeding, Anastomotic stenosis, Prolapse recurrence, and Urgency are presented as N (percentage), and Satisfaction index as Median (IQR)*.

We recorded a minimum of 48 months and a maximum of 60 months of follow-up, with a median of 57.3(16.9) months in the TST STARR+ group and 51.7(16.8) months in the PPH group. A total of 5 patients (3 in the PPH group and 2 in the TST STARR+ group) withdrew, and 120 patients completed the long-term follow-up. There were 43 patients in the TST STARR+ group and 77 in the PPH group.

By the end of the follow-up, a total of six patients had recurrent prolapse, 2 (4.7%) in the TST STARR+ group and 4 (5.2%) in the PPH group, and the difference was not statistically significant (*p* < 0.05). There was no significant difference in overall satisfaction, as shown in Table 4.

**Table 4:** Long term follow-up.

**Group**	**TST STARR +**	**PPH**	***χ^2^*/*z***	***P***
Number	43	77		
Follow-up months	57.3(16.9)	51.7(16.8)	1.802	0.074
Prolapse recurrence	2(4.7%)	4(5.2%)	0.017	0.896
Satisfaction index	10(1)	9(2)	3.006	0.003

*Follow-up months and Satisfaction index are presented as Median (IQR), and Prolapse recurrence as N (percentage)*.

## Discussion

Stapled hemorrhoidopexy (SH) (11), one of the most important innovations in proctology, offers the patients an effective and safe option, with less pain and fast recovery (12). For degree III hemorrhoids, PPH surgery is an effective treatment option to protect anal function (13, 14). A large number of studies have confirmed that, compared with conventional hemorrhoidectomy, PPH has the advantages of less postoperative pain, simple surgical operation, rapid recovery of work activity, and well protection of anal function (15, 16). Despite the short-term advantages of the PPH procedure, numerous studies have also shown that the recurrence rate after PPH is 3.4–45%, which is significantly higher than the recurrence rate after hemorrhoidectomy (17–22).The reason for the high recurrence rate after PPH is the failure to remove the hemorrhoid nucleus propria which has undergone pathological changes. The limited capacity of PPH anastomosis makes it unable to remove sufficient prolapsed tissue for some patients with severe degree III hemorrhoids with large internal rectal prolapse (23–27).

To reduce the recurrence rate after anastomosis in patients with hemorrhoidal prolapse, stapled transanal rectal resection (STARR) was developed in 2007 (27). Compared to the PPH procedure, the STARR procedure uses two anastomosing clutches and removes rectal prolapsed tissue more adequately, resulting in a better prognosis and a lower recurrence rate (28–30). However, the serious complications, such as rectovaginal fistulas, rectal wall hematomas, and serious pelvic infections limited its use in hemorrhoid surgery (31, 32).

TST STARR+ is a novel generation SH using high-volume devices equipped with innovative technology for the treatment of hemorrhoids with severe prolapse (10). As high-volume device, TST STARR+ allows the surgeon to regulate resection under direct view with a single device to remove a larger volume of cylindrical total circumferential rectal tissue. The revolutionary design of the anastomosis allows for a simpler procedure, in which a single anastomosis can adequately and safely remove the prolapsed tissue. Several clinical studies have confirmed that compared with the low-volume SH, TST STARR+ is safe and effective for severe hemorrhoidal prolapse, with the advantages of lowering long-term recurrence rate and reducing postoperative complications, such as pain, bleeding, wound complications, and constipation (17, 33, 34).

The Goligher classification, which is widely used in clinical practice, classifies hemorrhoids into 4° based on bleeding and prolapse. However, the classification does not consider the volume of prolapse in the rectum, which can also explain why some patients experience recurrence after PPH. We believe that a clear and reproducible criteria for the evaluation of internal prolapse are needed. Therefore, in addition to preoperative questioning and examination, it is more important to evaluate intraoperatively. Preoperative anesthesia can relax the pelvic floor muscles, which can then be positioned with CAD to more accurately assess the prolapsed entity in the rectum. A randomized trial showed that the incidence of re-prolapse within 1 year after PPH and STARR was 29.4 and 5.9%, respectively, in patients with hemorrhoids that prolapsed more than half of their CAD (27). CAD is a good predictor of re-prolapse, and patients with more than half of CAD who undergo PPH surgery are more likely to relapse (23). Particularly, the intraoperative positioning of the CAD has determined the emersion of asymmetric prolapse, which is important and necessary for selecting the patients (35).

In our study, the TST STARR+ group resected specimens with a median width of 4.8(1.1) cm and volume of 10.2(3) cm^3^ compared to 2.9(0.8) cm and 4.4(1.2) cm^3^ in the PPH group, showing that the TST STARR+ anastomosis can resect much larger specimens than the PPH anastomosis. Postoperative follow-up of at least 4 years revealed the recurrence of prolapse of 5.2% in the PPH group and 4.7% in the TST STARR+ group. There were no significant differences in short-term outcomes between the two procedures, including the number of intraoperative hemostatic needles, hospital stay, patient satisfaction, and common SH complications (36) such as postoperative pain, urgency, anastomotic stenosis. There were no serious AEs in either group, such as rectovaginal fistula, rectal perforation or anastomotic failure, which could be reduced by technical adjustments, as argued before (37). It is noteworthy to compare the postoperative bleeding dimension. The TST STARR+ procedure removed more tissue, which made it a higher risk of postoperative bleeding. However, the results of our study showed that the short-term efficacy of the two procedures did not differ significantly in the postoperative bleeding dimension. Twelve-month postoperative follow-up revealed that 3 patients (3.8%) in the PPH group reported minor bleeding, whereas no patients in the TST STARR+ group reported such a condition. We believe that, with a foot height of 4.2 mm and a closing range of 0.75–1.80 mm, the TST STARR+ anastomosis staple allows for safe resection and anastomosis of larger tissues (10).

At the same time, we found that grade III hemorrhoids were often associated with protruding external hemorrhoids, and residual external hemorrhoids after anastomosis surgery could lead to some clinical symptoms and patient dissatisfaction, sometimes requiring reoperation. In our study, external hemorrhoidectomy was performed on patients with combined significant external hemorrhoids. The follow-up results showed that no patients underwent reoperation for external hemorrhoid residue, and the postoperative satisfaction was high. Therefore, we believe that anastomosis surgery is necessary for the management of external hemorrhoids.

## Conclusions

In conclusion, the TST STARR+ procedure is more appropriate for patients with severe prolapse hemorrhoids greater than half of the CAD. The TST STARR+ procedure has the advantages of convenient to operate, rapid recovery, fewer AEs, and a lower long-term recurrence rate.

## Data Availability Statement

The raw data supporting the conclusions of this article will be made available by the authors, without undue reservation.

## Ethics Statement

The studies involving human participants were reviewed and approved by Changshu Hospital Affiliated to Nanjing University of Chinese Medicine. The patients/participants provided their written informed consent to participate in this study.

## Author Contributions

JW, XD, and JJ were responsible for the collection of clinical patients and the writing of the article. LJ and HH were responsible for the technical operation of the surgery and the revision of the article. All authors contributed to the article and approved the submitted version.

## Funding

The study was supported by the Special Project of Diagnosis and Treatment Technology for Key Clinical Diseases in Suzhou (LCZX202022) and Changshu Municipal Science and Technology Bureau Supporting Project (CS201925).

## Conflict of Interest

The authors declare that the research was conducted in the absence of any commercial or financial relationships that could be construed as a potential conflict of interest.

## Publisher's Note

All claims expressed in this article are solely those of the authors and do not necessarily represent those of their affiliated organizations, or those of the publisher, the editors and the reviewers. Any product that may be evaluated in this article, or claim that may be made by its manufacturer, is not guaranteed or endorsed by the publisher.
